# Do Alcohol Prevention Programs Influence Adolescents’ Drinking Behaviors? A Systematic Review and Meta-Analysis

**DOI:** 10.3390/ijerph18168524

**Published:** 2021-08-12

**Authors:** Mi-Kyoung Cho, Yoon-Hee Cho

**Affiliations:** 1 Department of Nursing Science, Chungbuk National University, Cheongju 28644, Korea; ciamkcho@gmail.com; 2 Department of Nursing, College of Nursing, Dankook University, Cheonan 31116, Korea

**Keywords:** alcohol prevention, drinking behavior, adolescent, meta-analysis

## Abstract

This study analyzed the effects of various alcohol prevention programs on the drinking behavior of adolescents. There were seven electronic databases used for the literature search. A systematic review and meta-analysis are employed for works published in Korean and English from January 2010 to April 2021, with strict inclusion criteria yielding 12 papers in the review. The type of alcohol prevention interventions included educational and motivational interventions. Six studies had more than 500 participants each, and five studies had more than 10 participating schools. The programs did not effectively reduce the frequency of drinking or binge drinking of adolescents but significantly reduced the amount of alcohol consumed. Based on the results of this study, when planning alcohol prevention programs for adolescents, it is necessary to adopt a multi-level approach, including the engagement of parents and the community.

## 1. Introduction

Several societies consider alcohol consumption to be a natural part of social life. However, drinking excessively can have considerably harmful consequences. Alcohol contains toxic substances and can be addictive; it acts as a major risk factor for premature death and disability when overused or abused [1]. According to a report evaluating the use of alcohol and the burden of disease across 195 countries, alcohol causes approximately 3 million deaths annually, accounting for 5.1% of the global disease burden [2]. Such harmful consequences of drinking tend to be worse in adolescents than adults. In particular, early drinking and binge drinking during adolescence can have negative health consequences throughout life, extending beyond the adolescent period [3].

According to an online survey on youth health conduct in Korea, 16.9% of boys and 13.0% of girls had consumed alcohol within the last month, and the proportion who engaged in dangerous drinking was 8.2% for boys and 7.5% for girls [4]. By comparison, for adults, 70.5% of men and 51.2% of women had consumed alcohol within the last month [4,5]. However, the proportion who engaged in dangerous drinking was 20.8% for men and 8.4% for women, indicating a much narrower gap in the rates for adolescents [4,5], particularly for female participants. Meanwhile, the starting age of underage drinking in Korea is decreasing, with a 2019 survey determining it as 13.2 years [4]. Such underage drinking has been reported to increase the likelihood of alcoholism and the possibility of becoming a problematic drinker in adulthood [6]. Therefore, drinking must be controlled before adulthood to increase the likelihood of appropriate drinking throughout life. In particular, it is imperative to provide preventative education by detecting problematic underage drinkers in adolescence to reduce drinking habits or delay the starting age of drinking.

Drinking in adolescence is influenced by various factors. In addition to biological factors (e.g., gender and age), family finances, and use of other substances, social and psychological factors (e.g., stress and depression), social group influences (e.g., relationships with peers, siblings, parents, teachers or schoolmates), the surrounding environment, laws related to alcohol, and mass media influence adolescents’ decision to drink [7,8,9,10]. Therefore, numerous past studies have attempted intervention and prevention programs to reduce drinking or the harmful consequences of alcohol consumption among adolescents. Such programs have attempted to analyze various interventions to modify the factors known to influence adolescents’ drinking, including social and psychological factors at the individual level, parents at the family level, and the surrounding environment at the school and community levels. However, there is a need for further analysis with stronger scientific evidence, as previous studies have produced contrasting or inconsistent results.

There exist reviews of studies that attempted to reduce or prevent adolescent drinking behavior, but these reviews are subject to limitations. Previous reviews addressed interventions for other substance use in addition to alcohol use [11,12,13] or alcohol use as part of multiple health risk behaviors [14]. In addition, only certain types of programs were selected and analyzed, such as parent-based programs [13,15,16], peer-based programs [17], brief interventions [18], and interventions using computers or the internet [11]. Additionally, as some studies included young adults [18], it was difficult to determine the effect of interventions on adolescents. Various programs can be tried to reduce or prevent alcohol use in adolescents, but the analysis of comprehensive programs may guide health providers to serve more effective programs.

Therefore, this study analyzed the effect of alcohol prevention programs on the drinking behavior of adolescents in Korea to calculate the merged effect size. In addition, by using sub-analysis based on various criteria, the study provides a basis for decision making when planning a drinking prevention program. Accordingly, a systematic review and meta-analysis was employed of studies published in Korean and English during 2010–2021, among other exclusion criteria that are discussed in the following section. The results could help the development of effective alcohol prevention interventions for adolescents in the future.

## 2. Methods

### 2.1. Research Design

This study is a systematic review and meta-analysis.

### 2.2. Inclusion Criteria

This study was conducted according to the systematic review handbook of the Cochrane Handbook for Systematic Reviews [19] and the systematic review reporting guidelines suggested by the Preferred Reporting Items for Systematic Reviews and Meta-Analyses (PRISMA) Group [20]. The “population, intervention, comparison, outcome, study design” was set before reviewing the literature.

The population (P) of this study comprised adolescents, the intervention (I) was an alcohol prevention intervention, the comparison (C) was with usual care, and the outcome (O) was drinking behaviors (frequency and amount of drinking, and frequency of binge drinking). Randomized controlled trials (RCT) and quasi-experimental designs were selected as the study design (SD). We searched for articles published from 1 January 2010 to 30 April 2021 in three Korean and four international electronic databases. Table 1 presents the eligibility criteria for this study. When repeated measurements were conducted in one study, we selected only papers that reported the values of the first post test, specific statistical values for the mean, the standard deviation, and the concrete number of samples, and were published in English or Korean.

### 2.3. Search Strategy

There were seven electronic databases used for the literature search: PubMed, the Cochrane Library, Cumulative Index to Nursing and Allied Health Literature (CINAHL), Medline, Korean Studies Information Service System (KISS), Research Information Sharing Service (RISS), and DBpia. In addition, the references of the searched papers and Google Scholar sites were manually searched to obtain comprehensive data. Regarding the searched keywords, the MeSH term, synonyms and related terms that depict “adolescent”, “alcohol”, “prevention”, and “alcohol drinking” were confirmed according to PICO through the MeSH DB in PubMed. The terms were modified and used according to the characteristics of each database. Search functions, such as MeSH term, text word, logical operator and truncation search, were utilized accordingly. The search protocol was registered in the PROSPERO International Prospective Register of Systematic Reviews (registration No. CRD42021249865 available at https://www.crd.york.ac.uk/prospero/#searchadvanced, accessed on 17 June 2021).

### 2.4. Quality Assessment

The quality of the papers was independently evaluated by two researchers (M.K.C. and Y.H.C.) using the Checklist for Randomized Controlled Trials and Checklist for Quasi-Experimental Studies from the Joanna Briggs Institute of Critical Appraisal Tools [21]. The Cochrane collaboration suggests using the RoB-2-0-tool for quality evaluation, but the RoB-2-0-tool is not suitable for evaluating quasi-experimental studies. Since RCT and quasi-experimental studies were included in this study, Joanna Briggs Institute’s quality assessment tool, which can evaluate RCT and quasi-experimental studies, was used rather than the RoB-2-0-tool. First, a pilot test was conducted on the research design of two articles according to the quality assessment tool to verify the agreement of scores. The researchers discussed the criteria items that did not match and agreed on the quality assessment criteria before independently performing the quality assessment. There were 13 quality assessment criteria items for RCTs and 9 for the quasi-experimental studies. The scores for each evaluation item were set as 0 (No, Unclear) or 1 (Yes).

### 2.5. Data Collection

A data search was performed by one researcher, and another researcher inputted the search formula in each database for the verification process. Two researchers (M.K.C. and Y.H.C.) conducted the process of examining the agreement of the extracted literature according to the inclusion and exclusion criteria for each stage. First, duplicated studies were removed by combining the list of literature searched from each database into one file. From the list of files excluding any duplicated works, the titles and the abstracts were reviewed again to confirm whether they satisfied the literature selection criteria; the reasons for excluding the literature were documented. When there were limitations in determining whether a particular study satisfied the selection criteria using the title or abstract, the original papers were reviewed to determine the selection. The same two researchers managed the bibliographic information of all studies equally, and the reasons for the excluded and the included works were documented at each stage. The studies that were selected in the final stage were extracted according to the author, publication year, publishing country, targeted research group, number of research participants, research design, type of intervention, intervention period, outcome variable and quality assessment score. These data were recorded into a coding table.

### 2.6. Data Analysis

The general characteristics of the research paper were presented in terms of frequency, percentage and average, and statistical analysis of the combined effect size, heterogeneity, Egger’s regression test, and Begg’s test was conducted using MIX 2.0 Pro (Ver. 2.0.1.6, BiostatXL, 2017, BiostatXL, CA, USA). Hedges’ g and 95% confidence intervals (CI) were calculated for the effect size, and the weight of each effect size was derived using the inverse of the variance [22]. The overall effect was calculated using the random effects model, which resets the weights by considering the variability among the participants of individual studies and the heterogeneity among studies. To assess heterogeneity of the target studies, Higgin’s I^2^ was calculated, which represents the variance among studies and specifically depicts the ratio of the actual variance to the total observed variance. The ratio was interpreted to be heterogeneous if I^2^ exceeded 50% [23]. The publication bias of the selected literature was verified by correcting the effect size using Egger’s regression test and Begg’s test [24].

## 3. Results

### 3.1. Data Extraction

**Figure 1:**
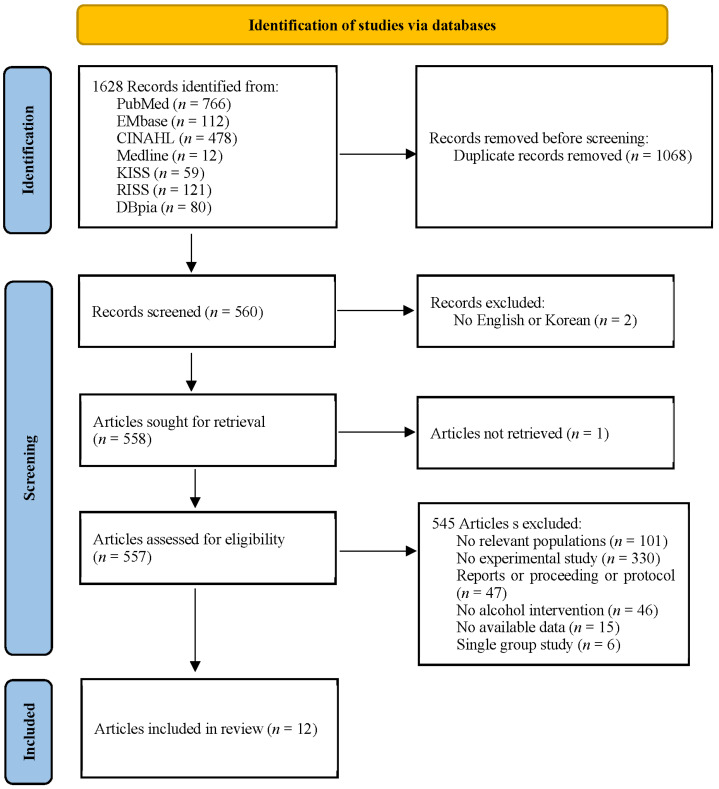
Diagram of the extraction flow.

The total number of studies returned from each database using the search strategy was as follows. There were 766 papers obtained from PubMed, 112 papers from EMBASE excluding PubMed from the Cochrane Library, 478 papers from CINAHL, 12 papers from Medline, 59 papers from KISS, 121 papers from RISS, and 80 papers from DBpia—a total of 1628 papers. After removing duplicated papers and papers that did not meet the inclusion criteria, a total of 12 papers were selected. There were three experimental groups analyzed in each study of Koning et al. [25] and Komro et al. [26], and two experimental groups in the study of Mckay et al. [27]. Therefore, in this review, each intervention performed in the experimental groups was separately divided into a–c and a, b for inclusion in this study (Figure 1).

### 3.2. Characteristics of the Study

There were seven papers published before 2015 and five papers published after 2015. Six studies were published in the United States and six studies were published in Europe. Five studies were targeted at those aged 16 years or older and six studies had more than 500 participants. Five studies were conducted in more than 10 schools. Nine studies examined individuals or schools, and three studies analyzed individuals, family, or communities. There were 11 RCTs and 1 quasi-experimental study. Three studies conducted educational interventions to improve knowledge and skills for alcohol prevention, and nine studies conducted motivational interventions. In the evaluation of the effect of the alcohol prevention program, seven repeated measures and five post-tests were performed. Seven studies had a quality assessment score of 10 or higher. The primary outcome variables of this study, drinking behavior, were frequency of drinking, amount of drinking and frequency of binge drinking. Frequency drinking was reported in 5 studies, amount of alcohol in 10 works, and frequency of binge drinking in 6 studies. Drinking knowledge and attitude, harm associated with alcohol use, or self-efficacy were reported in two studies and intention to drink alcohol in three studies (Table 2).

### 3.3. Methodological Quality

In the quality assessment, if the scores of the quality assessment items between the two researchers did not match because the results were described only in the abstract, not the text, the score was set as 0. The average score for methodological quality assessment of RCTs (11 articles) was 9.47 (range: 8 to 11), and that of quasi-experimental studies (1 article) was 8 points (Table 1). From the quality assessment items for the RCT studies, all 11 studies clearly described the following: “Was follow up complete and if not, were differences between groups in terms of their follow up adequately described and analyzed?”; “Were outcomes measured in the same way for treatment groups?”; and “Was the trial design appropriate, and any deviations from the standard RCT design (individual randomization, parallel groups) accounted for in the conduct and analysis of the trial?” Only four studies clearly described the following: “Were those delivering treatment blind to treatment assignment?” The quasi-experimental study clearly described all the items except “Were outcomes measured in a reliable way?” The quality level of the selected studies was relatively high. We concluded that there was no possibility of bias changing the conclusions of the results of the studies in the systematic review.

### 3.4. Effects of Alcohol Prevention Intervention on Drinking Behaviors

For the 12 selected studies, the Hedges’ g was calculated using the mean of the pre- and post-differences or post-test, the standard deviation of the difference or post-test, and the sample size of the two groups. The results are presented as a synthesis forest plot (Table 3). The overall effect on the frequency of drinking and binge drinking was small at 0.06 (95% CI: −0.57–0.64) and 0.29 (95% CI: −0.46–1.03), respectively. The overall effect size for frequency of drinking and binge drinking was very small and not statistically significant. The overall effect on the amount of drinking, one of the drinking behaviors, had a medium effect size of −0.46 (95% CI: −0.87 to −0.05). This decreased significantly after the alcohol prevention intervention (Z = −2.20, *p* = 0.028). In addition, the heterogeneity of the effect size was high at I^2^ = 98.2% [37]. Therefore, an exploratory exploration of the background of the effect size heterogeneity was deemed necessary, and sub-analysis was conducted according to the age and number of participants, number of schools, research design, type of program, target population, measurement, and quality assessment scores as the characteristics of the studies.

The sub-analysis results showed no difference in the frequency of drinking according to the characteristics of the studies, and the frequency of binge drinking significantly decreased only when the number of subjects was fewer than 500 (Hedges’ g: −0.21, 95% CI: −0.38 to −0.05). In the sub-analysis results, the amount of drinking decreased significantly among adolescents when the age of the subjects was under 16 years (Hedges’ g: −0.22, 95% CI: −0.38–0.07), there were more than 500 participants (Hedges’ g: −0.16, 95% CI: −0.30–0.03), the number of participating schools was more than 10 (Hedges’ g: −0.22, 95% CI: −0.39–0.05), and the intervention type was skill acquisition (Hedges’ g: −0.29, 95% CI: −0.40–0.18), when all individuals, families, and community were targeted (Hedges’ g: −0.28, 95% CI: −0.40–0.18). The amount of drinking among adolescents was not statistically significant according to the time of measurement and quality assessment score (Table 4).

### 3.5. Effects of Alcohol Prevention Intervention on Secondary Outcomes

The drinking behaviors were also measured using secondary outcome variables, such as knowledge and attitudes, harm associated with alcohol use, intention to drink alcohol, and self-efficacy in several studies (Table 5). Knowledge was measured in two studies; the effect size was medium at 0.54 (95% CI: 0.03–1.06) and increased to be statistically significant after the alcohol prevention intervention (Z = 2.06, *p* = 0.039). The effect sizes of the other variables were also merged but were not statistically significant.

### 3.6. Publication Bias Analysis

Egger’s regression test and Begg’s test were conducted to check the publication bias. Egger’s regression test is more suitable for parametric data than is Begg’s test because the former is a linear regression of the intervention effect estimates on their standard errors weighted by their inverse variance [38]. The results of Egger’s regression test for zero intercept showed an estimated intercept coefficient of −4.09 with a standard error of 3.30 (95% CI: −10.55–2.37); a *p*-value of 0.215 indicated no publication bias. Furthermore, the Y intercept was −4.09, which is less than 0, suggesting that the estimated intervention effect in the studies with fewer participants was less than that estimated in those with more participants. The results of Begg’s test for rank correlation revealed a tau b value of −0.22 and ties of 0, with no publication bias, similar to the results of Egger’s regression test (Z = −0.89, *p* = 0.371). Therefore, the degree of publication bias was acceptable, as the results of Egger’s regression test and Begg’s test confirmed that publication bias was not likely to change the findings of this study, namely that alcohol prevention interventions reduce alcohol consumption among adolescents.

## 4. Discussion

This study analyzed the effects of alcohol prevention programs applied to reduce the drinking behaviors of adolescents, to help develop more effective intervention plans. Adolescents generally drink less often than adults do but tend to consume more alcohol in one drinking session [39]. This is because adolescents have adolescent-specific sensitivity to alcohol during their developmental stages, tend to take greater risks, and are more influenced by their surroundings [39]. Therefore, unique drinking traits of adolescents must be considered in designing alcohol prevention programs for adolescents.

As a result of analyzing alcohol prevention programs conducted for adolescents to date, the programs could not effectively reduce the frequency of drinking or binge drinking of adolescents but were found to significantly reduce the amount of alcohol consumed. However, there was very little similarity in the results among studies. This finding is similar to the lack of consistent results in another review of alcohol misuse prevention programs for adolescents [16,40]. Nevertheless, in this study, the programs reviewed were found to be effective in reducing the amount of alcohol consumption in adolescents. Numerous previous studies have been conducted with a focus on primary prevention to delay the starting age of drinking. However, recent interventions have focused on reducing the hazards of drinking and have included adolescents who have already started drinking [27]. Such programs are believed to have contributed to the reduction in the quantity of alcohol consumed. Furthermore, the characteristics of the programs differed in their effectiveness in reducing the amount of drinking among adolescents.

Regarding the type of intervention, those involving skill acquisition were more effective in reducing the quantity of alcohol consumed than were single motivational interventions. This is similar to the findings of previous studies that reviewed primary prevention programs for alcohol misuse and reported psychosocial or developmental approaches; for example, life skill training and social skills have been shown to have some effect on alcohol misuse [40]. The researchers of such studies emphasized the surrounding individuals and the environment as factors that influence adolescent behavior [41].

The results of this study also found that interventions engaging families or the community, alongside adolescents, were more effective in reducing the amount of alcohol consumed than were interventions based on individuals. Adolescents generally spend most of their time at school. Schools are the most systematic and efficient places to take an educational approach to prevent the use of drugs in young people [42]. Therefore, numerous health programs for adolescents have been provided in schools [43]. However, Faggiano et al. found that school-based programs were effective in delaying the use of drugs but ineffective for adolescents who had already started using drugs [44]. Our results are consistent with such findings, which we believe to be the result of the focus on individual students in school-based programs. Therefore, in the future, alcohol interventions in schools should also involve families and communities.

According to previous studies, the drinking behaviors of adolescents were better controlled in studies that involved parents as intervention recipients than in studies that included only adolescents. Similar results were also found in reviews of parent-based interventions, in which parent-child programs were effective in reducing or preventing adolescent alcohol consumption [13,15]. This was more effective with younger adolescents [25], because the parents involved in the intervention controlled the behavior of adolescents through rule-setting restrictions [25,45]. Similarly, the results when analyzing the factors that influence drinking behavior in Korean adolescents revealed that family and surrounding environment exert more influence on the individual than do school factors [46]. Therefore, such aspects must be considered when establishing health programs for adolescents.

A study of American adolescents found differences in the prevalence of adolescents’ binge drinking according to the region [39]. This is because the norms regarding alcohol use, as well as alcohol regulation policies and the degree of coercion, differ in various regions [39]. The results may reflect the characteristics of adolescents who become easily affected by the social environment [47]. The results of the 2019 Youth Health Behavior Survey in Korea showed that it is not difficult for adolescents to purchase alcoholic beverages, with a success rate of over 70% (male students 74.9%; female students 71.5%) when high school students, who are legally prohibited from drinking alcohol, attempted to purchase alcohol at stores [4]. Such access to purchasing alcoholic beverages was found to be associated with high frequency and quantity of alcohol consumption [47]. Therefore, the development of programs for adolescents must be approached from multiple levels, including the atmosphere of the community and the health-related systems and policies. The programs were more effective in reducing the amount of alcohol consumption when applied to early adolescents. Therefore, when planning adolescent alcohol-related interventions, it is necessary to intervene at an early stage of developmental stage.

This study has several limitations. First, some of the studies included in the analysis had some methodological shortcomings. Some studies did not explicitly state the blinding of treatment delivery and the measurement of the outcome variables. Therefore, caution should be exercised when interpreting the results. Second, a sub-analysis was performed due to the large differences among effect sizes. As the number of studies included in the subgroup was small, the verification power was lowered; thus, caution should be adopted when interpreting the results. Third, in this study, Egger’s regression test and Begg’s test were performed to confirm the publication bias; the degree of publication bias was found to be acceptable. Because Egger’s regression test for publication bias lacks power to detect small study effects [48], researchers must be careful in interpreting the results of this test. Finally, the drinking behavior of adolescents was significantly affected by the social environment. Therefore, the social attitude toward alcohol in the country of study must be considered when interpreting the results.

## 5. Conclusions

This study conducted a systematic review and meta-analysis of the literature to analyze the effects of alcohol prevention programs on the drinking behavior of adolescents based on studies published between January 2010 and April 2021. Alcohol prevention programs were not found to effectively reduce the drinking frequency or the binge drinking behavior of adolescents. However, the programs significantly reduced the amount of drinking. According to the sub-analysis, interventions including skill training or social skills were more effective than motivational interventions to reduce the amount of alcohol consumed. Furthermore, interventions that included family or community were more effective than those involving individual students. Moreover, interventions given to early adolescents were more effective in reducing the amount of alcohol consumption compared to when not provided. Therefore, health providers should establish long-term programs with early interventions for early adolescents when planning alcohol prevention programs for adolescents. It will be necessary to adopt a multi-level approach, including the engagement of parents and community.

## Figures and Tables

**Table 1:** Eligibility criteria.

	Inclusion	Exclusion
Participants	Adolescents (13~18 years)	Studies of subjects participate in other studies that simultaneously affect drinking behavior Those who are diagnosed with alcoholism and are being treated
Interventions	Studies published from 1 January 2010 to 30 April 2021 Studies published in English or Korean Studies with mean, standard deviation, concrete sample size	Studies of subject is unable to voluntarily answer the questionnaire Studies in which the main effect of intervention is drug therapy Studies in which mean, standard deviation, and sample size of each group not accurately presented
Control	Usual care, comparative experiment	Participating in other interventions
Outcomes	Primary outcome is drinking behaviors (frequency of alcohol drinking, amount of alcohol drinking, frequency of binge drinking) Secondary outcomes are knowledge, attitudes, alcohol harm, intention to drinking, self-efficacy When serial interventions were performed, only the effect of the first intervention was coded for analysis	Studies that did not measure primary or secondary outcomes as an outcome variable
Study design	Quasi-experimental studies or RCT	Not quasi-experimental studies or RCT In the quasi-experimental study, a single group comparative study

**Table 2:** Characteristics of the included studies.

No.	Author (Year)	Country	Research Design	Target	Participants	Program	Duration	Outcome Variables	Quality Scores
1	Koning et al., (2014) [25]	Netherlands	cluster RCT	19 schools, individual or/and family	795 weekly drinking students (mean age: 12.66, SD = 0.49), E ^a^: 158, E ^b^: 251, E ^c^: 151, C: 235	PAS E ^a^: parent intervention, E ^b^: student intervention, E ^c^: combined intervention	≥6 month	drinking behavior (amount, frequency)	8
2	Mckay et al., (2012) [27]	Northern Ireland	non-randomized control longitudinal design	29 schools, individual	2187 (mean age: 13.84), E ^a^: 847, E ^b^: 574, C: 766	revised SHAHRP E ^a^: from teachers, E ^b^: from external facilitators (local drug and alcohol educators)	≥6 month	knowledge, attitudes, alcohol consumption, context of use, harm associated with own alcohol use and the alcohol use of other people	8
3	Zebregs et al., (2015) [28]	Netherlands	cluster RCT	12 school, individual	296 low educated adolescents 187 (age: 11–14), E: 161, C: 135	information about alcohol of narrative versus non-narrative form E: narrative information, C: non-narrative information	<6 month	knowledge, attitude towards alcohol, and intention to drink alcohol	10
4	Komro et al., (2017) [26]	Cherokee Nation	cluster RCT	6 communities (each with 1 high school), community/individual	1623 high school students (mean age: 14.9–15.2), E ^a^: 208, E ^b^: 224, E_3_: 603, C: 588	CMCA (community-organizing intervention targeting alcohol access), CONNECT (school-based universal screening and brief intervention) E ^a^: CMCA, E ^b^: CONNECT, E ^c^: combined intervention	≥6 month	current alcohol use, heavy episodic drinking	9
5	Armitage et al., (2014) [29]	North of England	RCT	1 school, individual	67 adolescents (mean age: 17.09, SD = 0.38), E: 32, C: 35	Brief Psychological Intervention (self-affirming implementation intention)	<6 month	alcohol intake, behavioral intention, self-efficacy	10
6	Doumas et al., (2017) [30]	USA	RCT	1 school, individual	221 high school seniors (mean age: 17.16, SD = 0.45), E: 116, C: 105	the eCHECKUP TO GO (brief, web-based personalized feedback intervention)	<6 month	drinking quantity, peak drinking quantity, frequency of drinking, problem drinking	11
7	D’Amico et al., (2012) [31]	USA	cluster RCT	16 middle schools, individual	8932 middle school students (mean age: 12.6), E: 4243, C: 4689	CHOICE (a voluntary after school program for younger adolescents)	<6 month	alcohol use, heavy drinking, perceived alcohol use, alcohol intentions, self-efficacy	10
8	Spirito et al., (2011) [32]	USA	RCT	PED, individual and family	97 students (mean age: 15.42–15.48), E: 41, C: 56	IMI + FCU E: IMI + FCU, C: IMI	≥6 month	drinking frequency, drinking quantity, frequency of high-volume drinking	9
9	Werch et al., (2011) [33]	USA	RCT	2 high schools, individual	451 public high school students (mean age: 17.08, SD = 0.82), E: 227, C: 224	brief integrative multiple behavior intervention	≥6 month	quantity x frequency of alcohol use	9
10	Gmel et al., (2012) [34]	Swiss	cluster quasi-randomized control trial	9 school, individual	668 secondary school students (age: 16–18), E: 338, C: 330)	brief group alcohol motivational intervention	<6 month	individual’s RSOD frequency, maximum number of drinks on a single occasion, and overall weekly consumption	10
11	Haug et al., (2017) [35]	Swiss	cluster RCT	11 schools, individual	1041 students (mean age: 16.8, SD = 1.6), E: 547, C: 494)	Mobile Coach Alcohol program	<6 month	frequency of RSOD, peak blood alcohol concentration, Overestimation of peer group drinking norms	11
12	Doumas et al., (2020) [36]	USA	RCT	2 schools, individual	283 students (mean age: 17.15, SD = 0.47), E: 159, C: 124	eCHECKUP TO GO	<6 month	frequency of alcohol use, peak drinking quantity, Normative beliefs about peer alcohol use, positive alcohol expectancies, Protective behavioral strategies	11

Notes. Superscripts a, b, and c indicate participants and program contents according to the type of program in the experimental group within one study. PED: pediatric emergency department, SD: standard deviation, E: experimental group, C: control group, RCT: randomized control trial, PAS: prevention of alcohol use in students, SHAHRP: school health and alcohol harm reduction project, CMCA: communities mobilizing for change on alcohol, CONNECT: individually delivered screening and brief intervention in schools, eCHECKUP TO GO: brief, web-based personalized feedback intervention, RAP: Reaching Adolescents for Prevention, IMI: individual motivational interview, FCU: Family Check-Up, RSOD: Risky single occasion drinking.

**Table 3:** Effect size of alcohol prevention interventions on drinking behaviors.

**Frequency of Drinking**									**Simple Forest Plot** **(Random Effect Model)**
**Study ID**	**Author (Year)**	***N***	**Hg**	**CI−**	**CI+**	**Z**	***p***	***w***	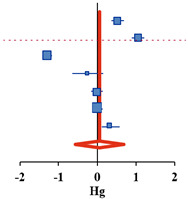
4^a^	Komro et al., (2017)	796	0.53	0.37	0.69	6.48	<0.001	14.4%	
4^b^	Komro et al., (2017)	812	1.05	0.89	1.21	12.68	<0.001	14.4%	
4^c^	Komro et al., (2017)	1191	−1.29	−1.41	−1.16	−20.24	<0.001	14.4%	
8	Spirito et al., (2011)	97	−0.25	−0.65	0.16	−1.20	0.231	13.7%	
10	Gmel et al., (2012)	668	−0.01	−0.16	0.15	−0.07	0.945	14.4%	
11	Haug et al., (2017)	1041	0.02	−0.10	0.14	0.32	0.749	14.4%	
12	Doumas et al., (2020)	283	0.35	0.12	0.59	2.92	0.003	14.2%	
Overall		3712	0.06	−0.57	0.69	0.19	0.853	100.0%	
**Amount of Drinking**									**Simple Forest Plot** **(Random Effect Model)**
**Study ID**	**Author (Year)**	***N***	**Hg**	**CI−**	**CI+**	**Z**	***p***	***w***	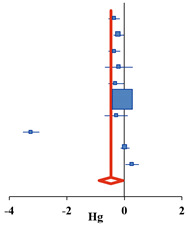
1 ^a^	Koning et al., (2014)	393	−0.35	−0.56	−0.15	−3.41	0.001	9.3%	
1 ^b^	Koning et al., (2014)	486	−0.19	−0.37	−0.02	−2.13	0.033	9.3%	
1 ^c^	Koning et al., (2014)	386	−0.34	−0.55	−0.14	−3.27	0.001	9.2%	
5	Armitage et al., (2014)	67	−0.19	−0.67	0.29	−0.78	0.437	8.2%	
6	Doumas et al., (2017)	221	−0.29	−0.55	−0.02	−2.12	0.034	9.1%	
7	D’Amico et al., (2012)	567	0.11	−0.05	0.28	1.32	0.186	9.3%	
8	Spirito et al., (2011)	8932	−0.06	−0.10	−0.02	−2.77	0.006	9.5%	
9	Werch et al., (2011)	97	−0.28	−0.69	0.12	−1.36	0.175	8.5%	
10	Gmel et al., (2012)	451	−3.24	−3.52	−2.96	−22.59	<0.001	9.0%	
12	Doumas et al., (2020)	668	0.03	−0.12	0.18	0.36	0.720	9.4%	
Overall		11,514	−0.46	−0.87	−0.05	−2.20	0.028	100.0%	
**Frequency of Binge Drinking**									**Simple Forest Plot** **(Random Effect Model)**
**Study ID**	**Author (year)**	***N***	**Hg**	**CI−**	**CI+**	**Z**	***p***	***w***	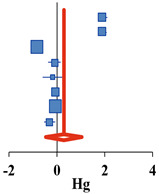
4 ^a^	Komro et al., (2017)	796	1.92	1.73	2.10	20.43	<0.001	12.6%	
4 ^b^	Komro et al., (2017)	812	1.90	1.72	2.08	20.72	<0.001	12.6%	
4 ^c^	Komro et al., (2017)	221	−0.11	−0.38	0.15	−0.82	0.410	12.4%	
6	Doumas et al., (2017)	567	0.05	−0.12	0.21	0.54	0.588	12.6%	
8	Spirito et al., (2011)	97	−0.19	−0.59	0.22	−0.91	0.363	12.1%	
10	Gmel et al., (2012)	668	−0.06	−0.21	0.09	−0.75	0.454	12.6%	
11	Haug et al., (2017)	1041	−0.04	−0.16	0.08	−0.63	0.527	12.6%	
12	Doumas et al., (2020)	283	−0.31	−0.54	−0.07	−2.55	0.011	12.5%	
Overall		3933	0.29	−0.46	1.03	0.76	0.450	100.0%	

Notes. Superscripts a, b, and c indicate participants and program contents according to the type of program in the experimental group within one study. *N*: sample size, Hg: Hedges’ g, CI: confidence interval, *w*: weight.

**Table 4:** The effect size of alcohol prevention interventions on drinking behaviors by intervention characteristics.

Variables	Characteristics	Subgroup	K	Study ID	*N*	Hg	95% CI		Z (*p*)	I^2^ (%)
							Lower Limit	Upper Limit		
Frequency of drinking	Age	<16	1	8	97	−0.25	−0.65	0.16	0.28 (0.231)	0.0
		≥16	4	4,10,11,12	3615	0.11	−0.58	0.79	0.31 (0.758)	99.2
	Number of participants	<500	2	8,12	380	0.08	−0.51	0.66	0.25 (0.799)	84.1
		≥500	3	4,10,11	3332	0.06	−0.72	0.84	0.15 (0.881)	99.4
	Number of schools	<10	4	4,8,10,12	2671	0.07	−0.73	0.86	0.16 (0.871)	99.2
		≥10	1	11	1041	0.02	−0.10	0.14	0.32 (0.749)	0.0
	Research design	RCT	5	4,8,10,11,12	3712	0.06	−0.57	0.69	0.19 (0.853)	99.0
	Type of program	Motivation	5	4,8,10,11,12	3712	0.06	−0.57	0.69	0.19 (0.853)	99.0
	Target population	Individual	4	4,10,11,12	3615	0.11	−0.58	0.79	0.31 (0.758)	99.2
		Family and community	1	8	97	−0.25	−0.65	0.16	0.28 (0.231)	0.0
	Measurement	repeated	2	4,8	1720	0.01	−1.22	1.24	0.02 (0.986)	99.5
		post	3	10,11,12	1992	0.10	−0.08	0.27	1.07 (0.284)	71.5
	Quality assessment scores	<10	2	4,8	1720	0.01	−1.22	1.24	0.02 (0.986)	99.5
		≥10	3	10,11,12	1992	0.10	−0.08	0.27	1.07 (0.284)	71.5
Amount of drinking	Age	<16	3	1,7,8	9824	−0.22	−0.38	−0.07	−2.82 (0.005)	76.0
		≥16	5	5,6,9,10,12	1690	−0.68	−1.87	0.50	−1.13 (0.259)	99.1
	Number of participants	<500	5	5,6,8,9,12	1119	−0.75	−2.12	0.63	−1.07 (0.286)	99.0
		≥500	3	1,7,10	10,395	−0.16	−0.30	−0.03	−2.41 (0.016)	77.5
	Number of schools	<10	6	5,6,8,9,10,12	1787	−0.62	−1.65	0.41	−1.18 (0.239)	98.9
		≥10	2	1,7	9727	−0.22	−0.39	−0.05	−2.54 (0.011)	81.0
	Research design	RCT	8	1,5,6,7,8,9,10,12	11,514	−0.46	−0.87	−0.05	−2.20 (0.028)	98.2
	Type of program	Skill acquisition	1	1	795	−0.29	−0.40	−0.18	−5.01 (<0.001)	0.0
		Motivation	7	5,6,7,8,9,10,12	10,719	−0.54	−1.17	0.10	−1.65 (0.098)	98.8
	Target population	Individual	6	5,6,7,9,10,12	10,622	−0.58	−1.28	0.13	−1.60 (0.109)	99.0
		Family and community	2	1,8	892	−0.29	−0.40	−0.18	−5.20 (<0.001)	0.0
	Measurement	repeated	3	1,8,9	1343	−0.88	−1.88	0.12	−1.72 (0.085)	98.9
		post	5	5,6,7,10,12	10,171	−0.02	−0.16	0.11	−0.35 (0.729)	64.9
	Quality assessment scores	<10	3	1,8,9	1343	−0.88	−1.88	0.12	−1.72 (0.085)	98.9
		≥10	5	5,6,7,10,12	10,171	−0.02	−0.16	0.11	−0.35 (0.729)	64.9
Frequency of Binge drinking	Age	<16	1	8	97	−0.19	−0.59	0.22	−0.91 (0.363)	0.0
		≥16	5	4,6,10,11,12	3836	0.35	−0.45	1.16	0.86 (0.391)	99.4
	Number of participants	<500	3	6,8,12	601	−0.21	−0.38	−0.05	−2.61 (0.009)	0.0
		≥500	3	4,10,11	3332	0.58	−0.45	1.61	1.09 (0.274)	99.6
	Number of schools	<10	5	4,6,8,10,12	2892	0.33	−0.59	1.26	0.71 (0.479)	99.4
		≥10	1	11	1041	−0.04	−0.16	0.08	−0.63 (0.527)	0.0
	Research design	RCT	6	4,6,8,10,11,12	3933	0.29	−0.46	1.03	0.76 (0.450)	99.3
	Type of program	Motivation	6	4,6,8,10,11,12	3933	0.29	−0.46	1.03	0.76 (0.450)	99.3
	Target population	Individual	5	4,6,10,11,12	3836	0.35	−0.45	1.16	0.86 (0.391)	99.4
		Family and community	1	8	97	−0.19	−0.59	0.22	−0.91 (0.363)	0.0
	Measurement	repeated	2	4,8	1720	0.70	−0.95	2.35	0.83 (0.406)	99.7
		post	4	6,10,11,12	2213	−0.10	−0.20	0.01	−1.84 (0.066)	26.8
	Quality assessment scores	<10	2	4,8	1720	0.70	−0.95	2.35	0.83 (0.406)	99.7
		≥10	4	6,10,11,12	2213	−0.10	−0.20	0.01	−1.84 (0.066)	26.8

Notes. K: number of studies, *N*: sample size, Hg: Hedges’ g, CI: confidence interval, RCT: randomized control trial.

**Table 5:** The effect size of alcohol prevention interventions on secondary outcome variables.

Variable	K	Study No.	*N*	Hg	95% CI		Z (*p*)	I^2^ (%)
					Lower Limit	Upper Limit		
Knowledge	2	2,3	2483	0.54	0.03	1.06	2.06 (0.039)	97.7
Attitudes	2	2,3	2483	−0.01	−0.10	0.08	−0.21 (0.835)	35.9
Harm associated with alcohol use	2	2,6	3174	0.01	−0.06	0.08	0.41 (0.683)	0.0
Intention to drink alcohol	3	3,5,12	646	0.07	−0.08	0.23	0.90 (0.366)	0.0
Self-efficacy	2	5,7	8999	0.02	−0.02	0.07	1.12 (0.263)	0.0

Notes. K: number of studies, *N*: sample size, Hg: Hedges’ g, CI: confidence interval.

## Data Availability

All data related to this study were available in the main manuscript. The search protocol was registered in the PROSPERO International Prospective Register of Systematic Reviews (registration No. CRD42021249865 available at https://www.crd.york.ac.uk/prospero/#searchadvanced, accessed on 17 June 2021).

## References

[1] World Health Organization (2020). Health Topics-Alcohol. https://www.who.int/health-topics/alcohol#tab=tab_1.

[2] Griswold M.G., Fullman N., Hawley C., Arian N., Zimsen S.R.M., Tymeson H.D., Venkateswaran V., Tapp A.D., Forouzanfar M.H., Salama J.S. (2018). Alcohol use and burden for 195 countries and territories, 1990–2016: A systematic analysis for the Global Burden of Disease Study 2016. Lancet.

[3] Spear L.P. (2015). Adolescent alcohol exposure: Are there separable vulnerable periods within adolescence?. Physiol. Behav..

[4] Korea Ministry of Education, Korea Ministry of Health and Welfare, Korea Disease Control and Prevention Agency (2019). The Statistics Report of the Fifteenth Korea Youth Risk Behavior Web-Based Survey.

[5] Korea Disease Control and Prevention Agency (2019). Korea Health Statistics 2018: Korea National Health and Nutrition Examination Survey (KNHANES VII-3).

[6] Gruber E., DiClemente R.J., Anderson M.M., Lodico M. (1996). Early Drinking Onset and Its Association with Alcohol Use and Problem Behavior in Late Adolescence. Prev. Med..

[7] Cho H.C. (2014). A meta-analysis on the factors related to adolescents’ alcohol use: From 1990 to 2012. Korean J. Youth Stud..

[8] Dir A.L., Bell R.L., Adams Z.W., Hulvershorn L.A. (2017). Gender Differences in Risk Factors for Adolescent Binge Drinking and Implications for Intervention and Prevention. Front. Psychiatry.

[9] Jackson N., Denny S., Ameratunga S. (2014). Social and socio-demographic neighborhood effects on adolescent alcohol use: A systematic review of multi-level studies. Soc. Sci. Med..

[10] Kim J.K., Kim G.H. (2013). Factors Affecting Drinking and Drinking Frequency among Korean Youth. Korean J. Youth Stud..

[11] Champion K.E., Newton N., Barrett E., Teesson M.R. (2012). A systematic review of school-based alcohol and other drug prevention programs facilitated by computers or the Internet. Drug Alcohol Rev..

[12] Das J.K., Salam R.A., Arshad A., Finkelstein Y., Bhutta Z.A. (2016). Interventions for Adolescent Substance Abuse: An Overview of Systematic Reviews. J. Adolesc. Health.

[13] Newton N.C., Champion K.E., Slade T., Chapman C., Stapinski L., Koning I., Tonks Z., Teesson M. (2017). A systematic review of combined student- and parent-based programs to prevent alcohol and other drug use among adolescents. Drug Alcohol Rev..

[14] Hale D.R., Fitzgerald-Yau N., Viner R.M. (2014). A Systematic Review of Effective Interventions for Reducing Multiple Health Risk Behaviors in Adolescence. Am. J. Public Health.

[15] Bo A., Hai A.H., Jaccard J. (2018). Parent-based interventions on adolescent alcohol use outcomes: A systematic review and me-ta-analysis. Drug Alcohol Depend..

[16] Hurley E., Dietrich T., Rundle-Thiele S. (2019). A systematic review of parent based programs to prevent or reduce alcohol consumption in adolescents. BMC Public Health.

[17] Ivaniushina V., Titkova V., Alexandrov D. (2019). Peer influence in adolescent drinking behaviour: A protocol for systematic review and meta-analysis of stochastic actor-based modeling studies. BMJ Open.

[18] Tanner-Smith E.E., Lipsey M.W. (2015). Brief Alcohol Interventions for Adolescents and Young Adults: A Systematic Review and Meta-Analysis. J. Subst. Abus. Treat..

[19] Cochrane Handbook of Systematic Reviews of Interventions. http://handbook.cochrane.org/.

[20] Moher D., Liberati A., Tetzlaff J., Altman D.G. (2009). Preferred reporting items for systematic reviews and meta-analyses: The PRISMA statement. BMJ.

[21] JBI Checklist for Case Control Studies. https://jbi.global/critical-appraisal-tools.

[22] Bax L. MIX 2.0. Professional Software for Meta-Analysis in Excel. https://www.meta-analysis-made-easy.com.

[23] Borenstein M., Hedges L.V., Higgins J.P.T., Rothstein H.R. (2009). Introduction to Meta-Analysis.

[24] Mavridis D., Salanti G. (2014). How to assess publication bias: Funnel plot, trim-and-fill method and selection models. Evid. Based Ment. Health.

[25] Koning I.M., Lugtig P., Vollebergh W.A. (2014). Differential effects of baseline drinking status: Effects of an alcohol prevention program targeting students and/or parents (PAS) among weekly drinking students. J. Subst. Abus. Treat..

[26] Komro K.A., Livingston M.D., Wagenaar A.C., Kominsky T.K., Pettigrew D.W., Garrett B.A., Cherokee Nation Prevention Trial Team (2017). Multilevel Prevention Trial of Alcohol Use among American Indian and White High School Students in the Cherokee Nation. Am. J. Public Health.

[27] McKay M.T., McBride N.T., Sumnall H.R., Cole J.C. (2012). Reducing the harm from adolescent alcohol consumption: Results from an adapted version of SHAHRP in Northern Ireland. J. Subst. Use.

[28] Zebregs S., Putte B.V.D., De Graaf A., Lammers J., Neijens P. (2015). The effects of narrative versus non-narrative information in school health education about alcohol drinking for low educated adolescents. BMC Public Health.

[29] Armitage C.J., Rowe R., Arden M.A., Harris P.R. (2014). A brief psychological intervention that reduces adolescent alcohol consumption. J. Consult. Clin. Psychol..

[30] Doumas D.M., Esp S., Flay B., Bond L. (2017). A Randomized Controlled Trial Testing the Efficacy of a Brief Online Alcohol Intervention for High School Seniors. J. Stud. Alcohol Drugs.

[31] D’Amico E.J., Tucker J.S., Miles J.N.V., Zhou A.J., Shih R.A., Green H.D. (2012). Preventing Alcohol Use with a Voluntary After-School Program for Middle School Students: Results from a Cluster Randomized Controlled Trial of CHOICE. Prev. Sci..

[32] Spirito A., Sindelar-Manning H., Colby S.M., Barnett N.P., Lewander W., Rohsenow D.J., Monti P.M. (2011). Individual and Family Motivational Interventions for Alcohol-Positive Adolescents Treated in an Emergency Department. Arch. Pediatr. Adolesc. Med..

[33] Werch C.E., Bian H., Carlson J.M., Moore M.J., DiClemente C.C., Huang I.-C., Ames S.C., Thombs D., Weiler R.M., Pokorny S.B. (2010). Brief integrative multiple behavior intervention effects and mediators for adolescents. J. Behav. Med..

[34] Gmel G., Venzin V., Marmet K., Danko G., Labhart F. (2012). A quasi-randomized group trial of a brief alcohol intervention on risky single occasion drinking among secondary school students. Int. J. Public Health.

[35] Haug S., Castro R.P., Kowatsch T., Filler A., Dey M., Schaub M.P. (2017). Efficacy of a web- and text messaging-based intervention to reduce problem drinking in adolescents: Results of a cluster-randomized controlled trial. J. Consult. Clin. Psychol..

[36] Doumas D.M., Esp S., Turrisi R., Bond L., Flay B. (2020). Efficacy of the eCHECKUP TO GO for High School Seniors: Sex Differences in Risk Factors, Protective Behavioral Strategies, and Alcohol Use. J. Stud. Alcohol Drugs.

[37] Melsen W.G., Bootsma M.C.J., Rovers M.M., Bonten M.J.M. (2014). The effects of clinical and statistical heterogeneity on the predictive values of results from meta-analyses. Clin. Microbiol. Infect..

[38] Lin L., Chu H., Murad M.H., Hong C., Qu Z., Cole S.R., Chen Y. (2018). Empirical Comparison of Publication Bias Tests in Meta-Analysis. J. Gen. Intern. Med..

[39] Chung T., Creswell K.G., Bachrach R., Clark D.B., Martin C.S. (2018). Adolescent Binge Drinking. Alcohol Res. Curr. Rev..

[40] Foxcroft D.R., Tsertsvadze A. (2012). Universal alcohol misuse prevention programmes for children and adolescents: Cochrane systematic reviews. Perspect. Public Health.

[41] Foxcroft D.R., Ireland D., Lister-Sharp D.J., Lowe G., Breen R. (2003). Longer-term primary prevention for alcohol misuse in young people: A systematic review. Addiction.

[42] Faggiano F., Vigna-Taglianti F., Versino E., Zambon A., Borraccino A., Lemma P. (2005). School-based prevention for illicit drugs’ use. Cochrane Database Syst. Rev..

[43] Caria M.P., Faggiano F., Bellocco R., Galanti M.R. (2011). Effects of a School-Based Prevention Program on European Adolescents’ Patterns of Alcohol Use. J. Adolesc. Health.

[44] Faggiano F., Galanti M.R., Bohrn K., Burkhart G., Vigna-Taglianti F., Cuomo L., Fabiani L., Panella M., Perez T., Siliquini R. (2008). The effectiveness of a school-based substance abuse prevention program: EU-Dap cluster randomised controlled trial. Prev. Med..

[45] Cuijpers P., Jonkers R., De Weerdt I., De Jong A. (2002). The effects of drug abuse prevention at school: The ‘Healthy School and Drugs’ project. Addiction.

[46] Kim E., Bang S.A., Seo E. (2019). A Study on Factors Influencing Youth Drinking Using Binomial Logistic Regression. J. Korean Soc. Comput. Info..

[47] Holmila M., Karlsson T., Warpenius K. (2010). Controlling teenagers’ drinking: Effects of a community-based prevention project. J. Subst. Use.

[48] Rodgers M.A., Pustejovsky J.E. (2021). Evaluating meta-analytic methods to detect selective reporting in the presence of dependent effect sizes. Psychol. Methods.

